# iMarmot: an integrative platform for comparative and functional genomics of marmots

**DOI:** 10.1186/s12864-020-6697-7

**Published:** 2020-03-30

**Authors:** Baoning Liu, Liang Bai, Qingqing Yu, Fang Hu, Jing Wu, Sihai Zhao, Rong Wang, Weirong Wang, Yuanqing Tao, Jianglin Fan, Enqi Liu

**Affiliations:** 1 Laboratory Animal Center, Xi’an Jiaotong University Health Science Center, No.76, Yanta West Road, Xi’an, 710061 Shaanxi China; 2 Research Institute of Atherosclerotic Disease, Xi’an Jiaotong University Cardiovascular Research Center, Xi’an, 710061 Shaanxi China; 3 Laboratory Animal Center, Ningxia Medical University, Yinchuan, 750004 Gansu China; 4 Qinghai Institute for Endemic Disease Prevention and Control, Xining, 811602 Qinghai China; 5 Department of Molecular Pathology, Faculty of Medicine, Interdisciplinary Graduate School of Medicine, University of Yamanashi, Yamanashi, 409-3898 Japan

## Abstract

**Background:**

Marmots are large Holarctic rodents with unique biological features, making them potential animal models in various research fields. Due to the rapid accumulation of the genetic data in marmots, a highly integrative database is urgent needed.

**Description:**

iMarmot is freely available on the web at http://www.marmotdb.org/ and currently contains the biological information of 14 marmots, genomic sequence of 6 marmots, syntenic relationship and orthologs among 3 marmots, and expression profiles of several hibernators and plague hosts. To assist with the genomic and transcriptomic analysis, we also integrated a set of analysis and visualization tools, such as KEGG or GO enrichment analysis, PCA, Blast, Muscle, GeneWise, Lastz, and JBrowse. Particularly, one DEGs (differentially expressed genes) module has been implemented in this database to visualize the gene expression changes in hibernators and plague hosts.

**Conclusion:**

This database will provide comprehensive information and analysis platform for researchers interested in understanding the biological features of marmots.

## Background

Marmots have been known since antiquity and firstly recognized by Thorington and Hoffman [1]. They are placed in the genus *Marmota*, family *Sciurid* with 14 extant species [1]. Most of them are living in social colonies and use loud whistles to communicate with one another. They prefer to habitat in the rock mountains or alpine meadows in the Holarctic, where there is a long and cold winter time without food [2–4]. To cope with the harsh environment, marmots have developed the capability of hibernation in burrows during winter. Recently, global climate changes are affecting fauna and flora in various ways. Marmots living in alpine might encounter problems caused by rising of the timberline or from the abandonment of pastures. For instance, Vancouver Island marmot (*Marmota vancouverensis*)*,* a typical alpine-dwelling marmot, has become one of the most endangered mammals in the world [5]. An international marmot network of scientists and managers has been established to work on the conservation issues of these species since 2008 (http://www.cons-dev.org/marm/MARM/EMARM/framarm/framarm.html). Meanwhile, plagues prevalence in some marmots were frequently reported to co-occur with sporadic epidemic in people all over the world, which also give rise to special supervision to the population fluctuations and epizootic activity of marmots [6–8]. Therefore, not only sociobiologists, conservationists and epidemiologist pay high attention to marmots, but also physiologists, who consider marmots as excellent animal models for the research of hibernation mechanism with possible applications to human medicine [4, 9].

Thanks to the development of sequencing technologies, large volumes of genomic and transcriptomic data have been generated. In 2015, the Alpine marmot (*Marmota marmota*) draft genome sequence was firstly released in the GenBank database of National Center for Biotechnology Information (NCBI) (accession number: GCA_001458135.2). In 2018, the Yellow-bellied marmot (*Marmota flaviventris*) draft genome was also released (accession number: GCA_003676075.2). Meanwhile, our group has assembled and published the genome sequence of the Himalayan marmot (*Marmota himalayana*) and re-sequenced the whole genomes of another four marmots, including Mongolia marmot (*Marmota sibirica*), Gray marmot (*Marmota baibacina*), Long-tailed marmot (*Marmota caudata*) and Yellow-bellied marmot in 2019 [10]. Particularly, RNA-seq or microarray data from a broad range of mammal hibernators and plague hosts provide novel insight into understanding the special biological features of marmots, especially the molecular mechanism of hibernation [10–16]. However, there is no available database to save, exploit, analyze and distribute these large-scale datasets. To facilitate the usage and application of these genomic, transcriptomic data and comparative results, we have developed the marmot database (iMarmot: www.marmotdb.org) as a highly integrative information platform with online analysis tools. We believe that the web-based platform with user-friendly interface and useful functions of will undoubtedly help the researches from a broad range of ecological and biomedical fields.

## Construction and content

### Database implementation

The iMarmot was implemented in Linux operation system and built using Akka HTTPServer (web server) and MySQL (database server). MySQL Database Management System (version 5.7.23) was used to process and organize all the data. The Twirl template engine and Bootstrap were applied to design and execute the interface components of the website. The query function was enforced based on Slick middleware tier. Boxplot, Barplot, Scatterplot and Heatmap plot were produced by Plotly 2.6.0 and Highcharts 5.0.12.

### Biological features of marmots

**Fig. 1:**
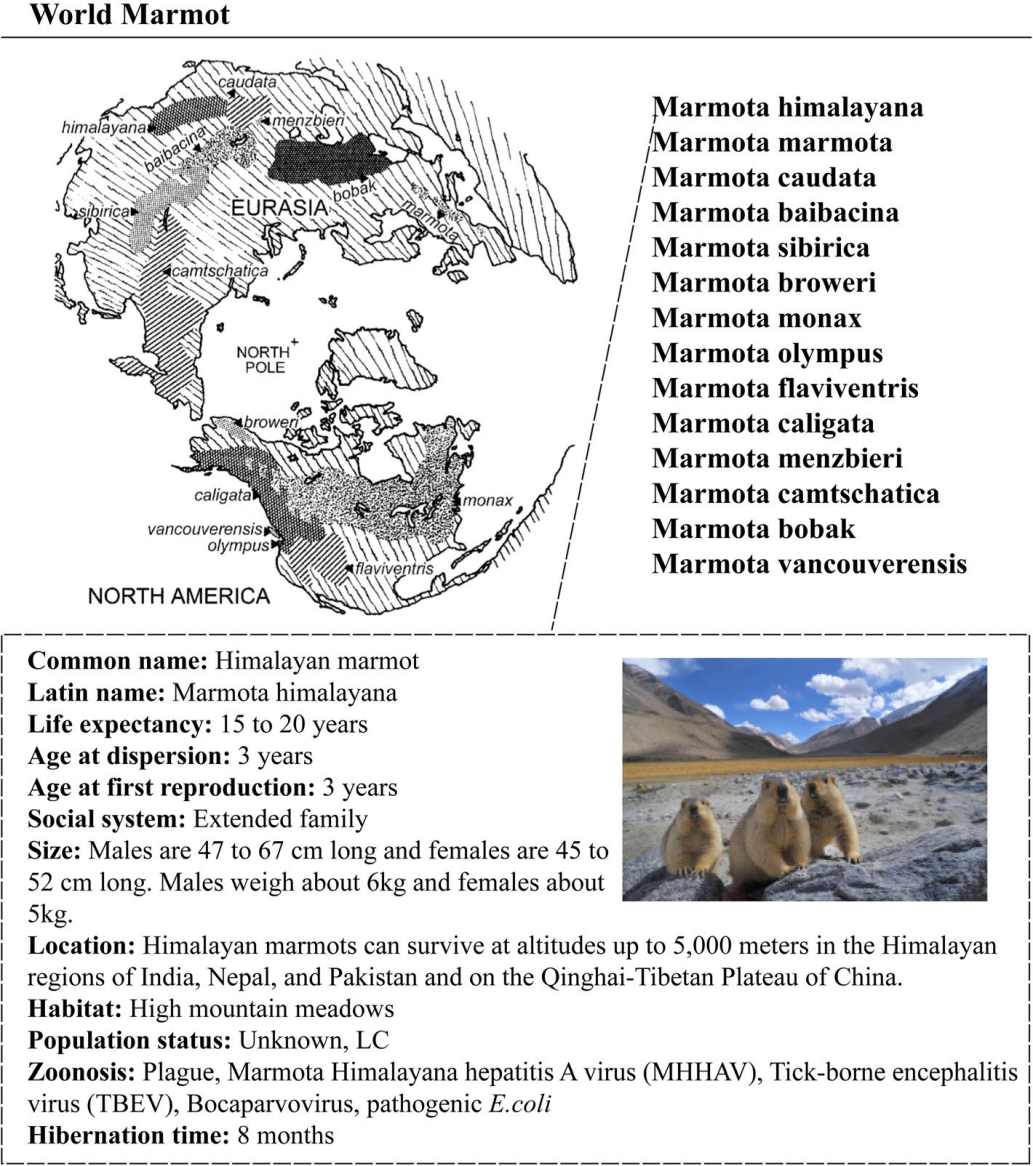
The world locations and biological features of marmots curated from public resources. The map of geographic distribution of 14 *Marmota* species (top left) was derived from Cardini, 2003 [2]. The Himalayan marmot image (bottom right) was obtained from China Foto Press (Tianjin) Image Technology Company Limited. We have obtained the appropriate copyright permissions to use and adapt them. Meanwhile, we would like to thank their contribution to our work

We have collected and integrated the species information of 14 extant marmots from the international marmot network, ADW (animal diversity web), IUCN Redlist (International Union for Conservation of Nature Redlist) and published literatures. For enhancing the readability of the integrated information, detailed data about marmots were shown in the categories of size, habitat, location, population status, zoonosis, hibernation time, social system, dispersion and reproductive age (Fig. 1).

### Genome sequence and annotation

The iMarmot serves as a public warehouse to annotate, release and update the whole genome sequence of marmots. At present the iMarmot possesses three de novo genome assemblies, including Himalayan marmot, Alpine marmot and Yellow-bellied marmot. The assembly sequence of Alpine marmot and Yellow-bellied marmot were downloaded from the GenBank assembly database of NCBI, while the assembly of Himalayan marmot was derived from our previous work [10]. Totally, 21,609 protein-coding genes have been predicted for Himalayan marmot, 23,923 for Alpine marmot and 24,575 for Yellow-bellied marmot. A uniform protocol was applied to comprehensively annotate the function of these putative protein-coding genes. Functions of these protein-coding genes were assigned according to the best match of the alignments against various protein databases using BLASTP (*e*-value = 1e^− 5^) [17], including the nonredundant protein (Nr) database, Swiss-Prot database and TrEMBL. Furthermore, unigenes were searched against the NCBI nonredundant nucleotide sequence (Nt) database using BLASTN [17] with a threshold of *e*-value = 1e^− 5^. InterProScan (v4.3) [18] was used to collect the protein domain information and Gene Ontology (GO) terms [19]. Meanwhile, all protein-coding genes were aligned against KEGG (Kyoto Encyclopedia of Genes and Genomes), then KAAS (KEGG Automatic Annotation Server) was used for the KEGG pathway annotation [20]. As a result, 19,470 protein-coding genes were annotated for Himalayan marmot, 20,832 for Alpine marmot genome and 20,217 for Yellow-bellied marmot genome. Furthermore, 290 KEGG pathways and 13,334 GO terms were involved in the Himalayan marmot genome, 293 KEGG pathways and 11,389 GO terms in the Alpine marmot genome, 290 KEGG pathways and 11,400 GO terms in the Yellow-bellied marmot genome.

### Synteny blocks and orthologous genes

**Fig. 2:**
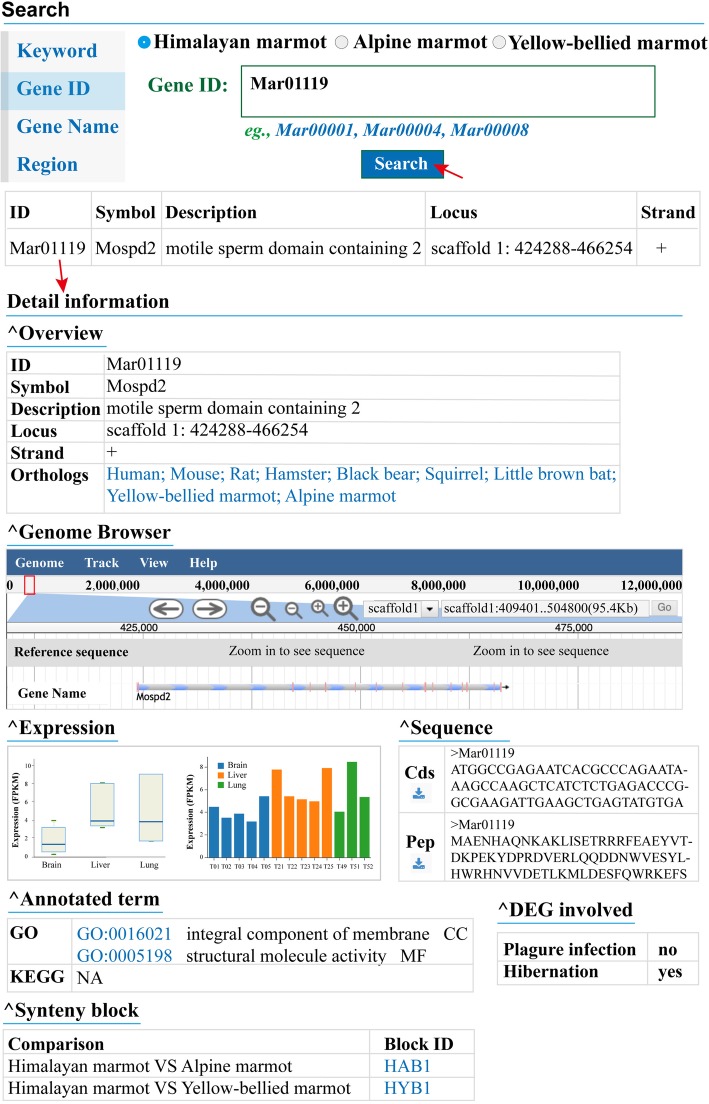
Overview of the ‘Search’ section of iMarmot database. The gene feature page contains several sections, including Genome Browser, Expression, Sequence, Annotated term, Synteny blocks, etc. Mar01119 was taken as an example

**Fig. 3:**
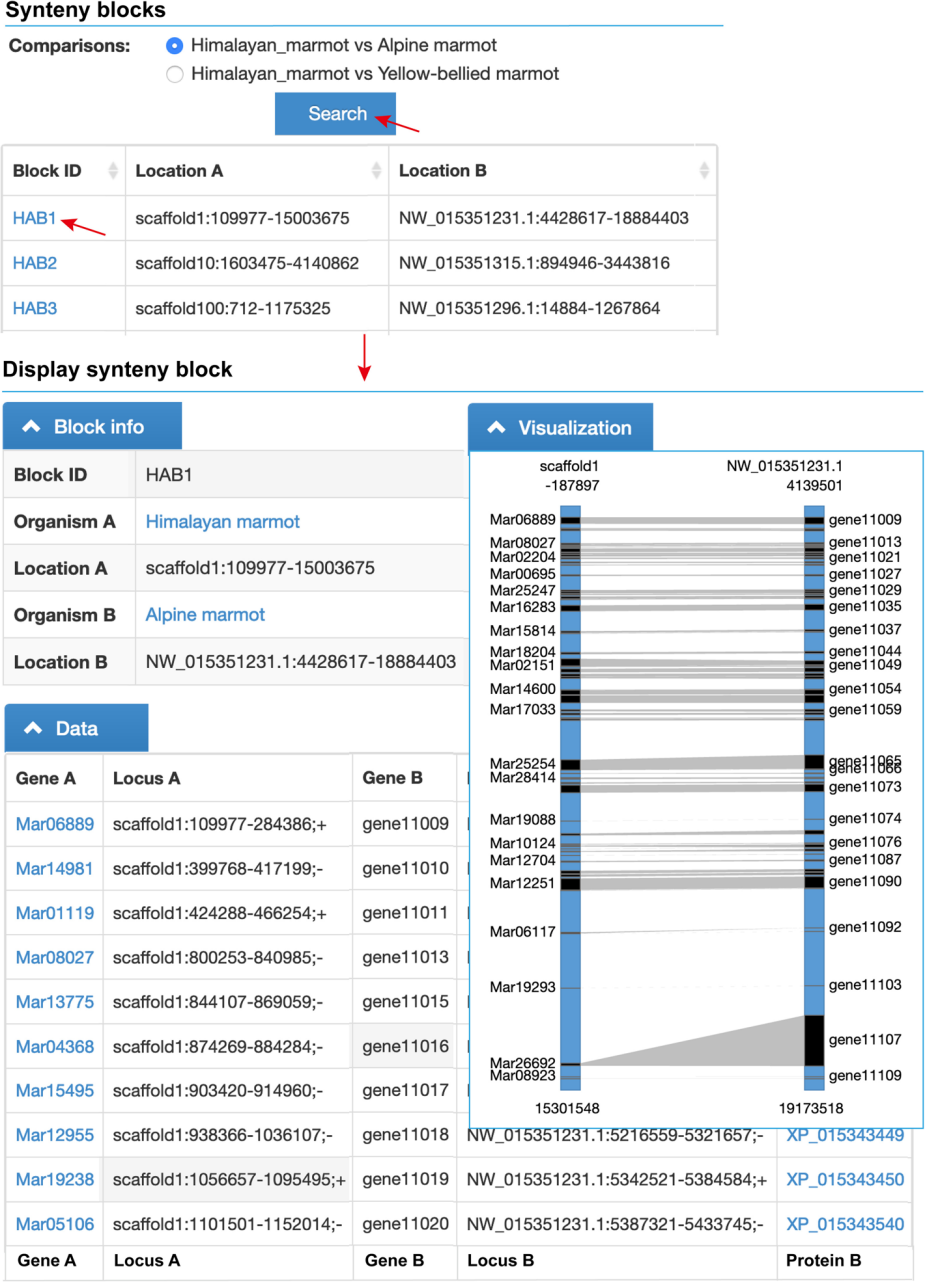
Overview of the synteny block. The visualization of selected synteny block is shown according to the homologous gene pairs, which are connected by grey lines

The synteny blocks and homologous genes were detected for the genome comparisons of Himalayan marmot versus Alpine marmot and Himalayan marmot versus Yellow-bellied marmot. All of the protein sequence were firstly BLASTP against each other with an *e*-value cut-off of 1e^− 5^ [17]. Based on these results, MCScanX was applied to determine the synteny blocks by default parameters [21]. Totally, 949 synteny blocks and 14,397 orthologous genes have been identified for the comparison of Himalayan marmot versus Alpine marmot, 951 synteny blocks and 13,935 orthologous genes for comparison of Himalayan marmot versus Yellow-bellied marmot. Each synteny block was embedded as a ‘Synteny’ subsection in its related gene feature (Fig. 2). The database users could view the detailed information of all the orthologous gene pairs located in the synteny region in a further linked web page (Fig. 3).

### Genomic variations

The iMarmot database currently contains high quality SNP data of 5 marmots, including an albinistic Himalayan marmot, Long-tailed marmot, Gray marmot, Yellow-bellied marmot, and Mongolian marmot. The raw sequence data of 5 marmots were derived from our previous work. Clean reads were mapped to the Himalayan marmot reference genome using BWA [22] and sorted by SAMtools [23]. We then marked duplicates with Picard tools (v1.94). SNP were then called following a standard procedure using the Genome Analysis Toolkit (GATK 3.0) with default parameters [24]. Variants were removed if they meet any of the following criteria: 1) an overall quality score of < 30; 2) a mapping quality score of < 60; 3) a phred-scaled *p*-value > 60; 4) a variant quality by depth score < 2; 5) genotype quality score < 5. In total, 24,192,293 SNPs of 5 marmots were retained. Users can visualize SNPs annotated in the Himalayan marmot genome in JBrowse and access each marmot’s SNPs in Download page.

### Expression data of hibernators and plague hosts

To understand the biological features of marmots, we have collected the expression profiles derived from RNA-seq or microarray data of several hibernators and plague hosts obtained from the Gene Expression Omnibus (GEO) database of NCBI. For RNA-seq data, the SRA toolkit (version 2.9.6) was used to convert the data from SRA to FASTQ format and then applied cutadapt (version 1.18) [25] with the following criteria for the quality control: 1) remove adaptors; 2) base quality > 20. After obtaining the high-quality data, mRNA sequencing reads were analyzed by unified protocol. First of all, HISAT was used to map the raw RNA-seq reads to their corresponding genomes [26]. Subsequently, the alignments were delivered to StringTie [27], which would assemble the full-length transcripts representing multiple splice variants for each gene locus. The merged full-length transcripts were given to StringTie again for obtaining better quantification of transcript richness. At last, Ballgown obtained all transcripts and abundances from StringTie and classified them according to the sample group [28]. For microarray, the raw data was downloaded from the GEO repository. Then R package Limma was applied to perform the data standardization and assess the differential expression via pairwise comparison. If multiple probes existed for one gene, the mean expression of these probes is considered as the expression of this gene. The criterion is *p*-value less than 0.05. Totally, 6 plague infection projects and 14 hibernation projects were retained, containing 536 samples, covering 14 tissues and 10 species.

## Utility and discussion

### Search and browse

To facilitate the query for gene information, we built the index for various types of annotation data, such as gene symbol, GO terms and KEGG pathway, and provided a basic search form for the Himalayan marmot genome, Alpine marmot and Yellow-bellied marmot. The users only need to input a single or batch of gene IDs, gene symbols or genome regions for retrieving the specific gene information. Each gene has a detailed information page, which is divided into subsections including the general information, gene structure implemented in JBrowse, sequence of protein and CDS, annotated term, expression pattern, as well as synteny block (Fig. 2). Moreover, users could also browse the GO or KEGG category and synteny blocks for the Himalayan marmot, Alpine marmot and Yellow-bellied marmot. Genes within each annotated category or synteny block will be displayed and further linked to Genome browser. In addition, we also provided a search function aside the main menu, which allows global keyword queries against all the records stored in the database.

### Genome browser

In iMarmot database, we have implemented JBrowse, a commonly used Genome browser, to display the genome sequences, gene models, genomic variations [29]. Currently, de novo assemblies of 3 marmots and genomic variations of 5 marmots were imported into JBrowse [29]. Particularly, there is a Himalayan marmot genome track embedded in the Genome Browser subsection in the gene feature page. This subsection provides a graphical and informative view of the gene structure of the searched gene model (Fig. 2). In addition, the JBrowse has several supporting tracks of non-coding RNA, pseudogene, repetitive sequence and GC content for the Himalayan marmot. In addition, JBrowse also provides access to easily add the genomic information of other marmots.

### DEGs visualization

In order to infer the molecular mechanism underlying the biological features of marmots, we have developed the DEGs module for visualization the differential expression profiles derived from the transcriptome data of several hibernators and plague hosts. Users can easily filter the DEGs within one project by tissues, comparisons, up or down regulation status, fold change value, and *p*-value. The result page of differential expression analysis contains the parameters used for statistical analysis, project description, significant DEGs list, and linkages to download file containing the differential expression analysis results. The file format of DEGs directly conform to the request of Online tools, such as enrichment analyses, PCA and correlation heatmap.

### Online tools

Numerous candidate genes were proposed in the comparative genomic and transcriptomic studies. The designating of those genes into specific functional categories would definitely facilitate the perception of the potential molecular mechanism under certain pathophysiological status. By enrichment analysis, we are able to detect the significantly overrepresented functional classes in a given gene list, which provide a useful tool to infer the major influenced functional categories or biochemical pathways in organisms with specific experimental treatments. In the iMarmot database, we have embedded the two commonly used tools: GO and KEGG tools, which facilitate the users to find the significantly enriched GO terms and KEGG pathways by uploading an interesting gene list. The ‘GO tool’ was executed by involving the GO::TermFinder Perl module. The ‘Pathway tool’ was operated based on the PathwayTools [30]. The hypergeometric distribution test was applied to determine the significance of enriched gene class in either GO or KEGG tool [31]. We have also integrated some other frequently used genomic and transcriptomic tools, such as Blast [17], Muscle [32], Lastz [33], Genewise [34], PCA, hCluster and correlation heatmap. Users can upload their own data, fill in the parameters and submit the task. Then a result page will appear automatically.

### Other functions

In the Download page, we provide linkages for batch data download. The users can directly download all the processed data, including the whole genome sequence, protein and CDS sequences, gene function annotation, SNP data and expression profile data. Meanwhile, iMarmot users are also welcome to share their own data with us. In the Submit page, users could find the format requirements of each data type that can be integrated to the database. In addition, a “How to” page and a “FAQ” page give the instructions of main functions to the iMarmot users and help them easily manage the database.

## Conclusions

**Fig. 4:**
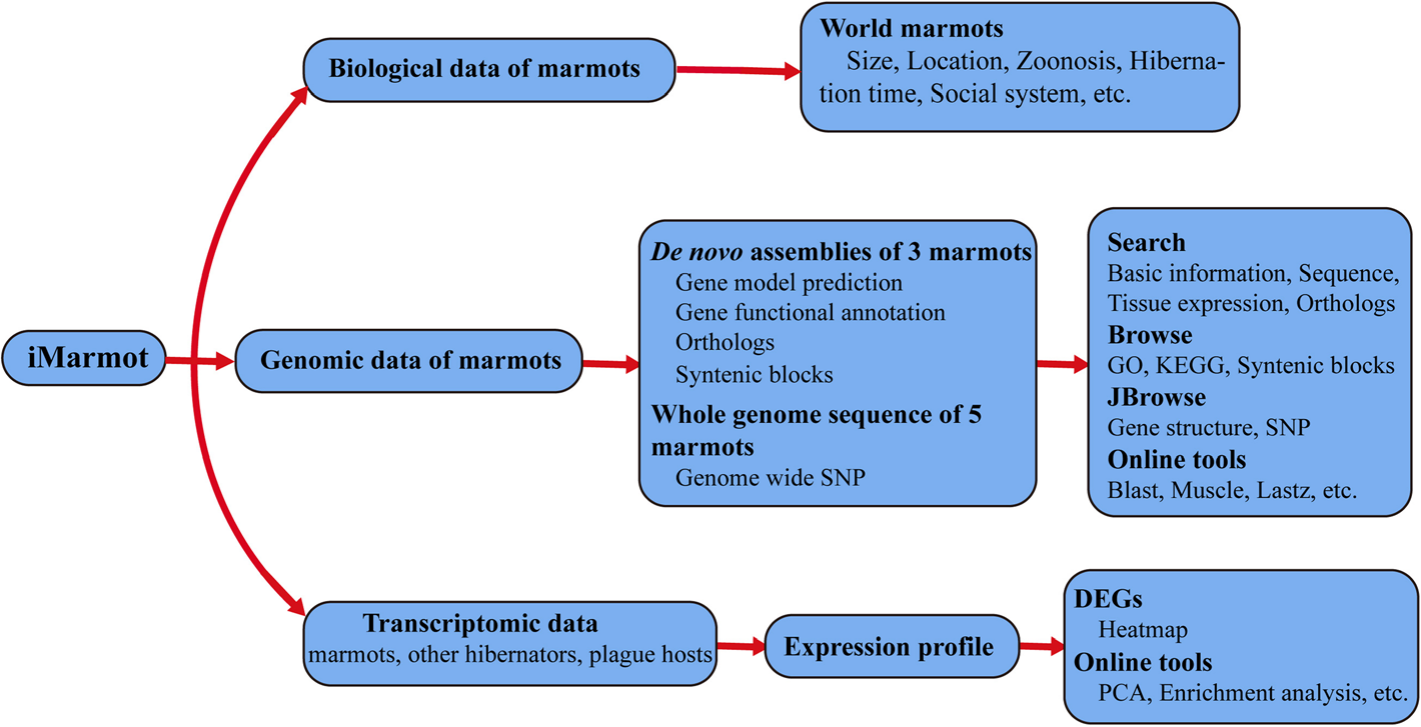
Data and feature diagram of iMarmot database. The iMarmot database incorporates the biological, genomic and transcriptomic data of marmots. All the data are manually curated and integrated. Various tools are embedded in the webpage to retrieve and analyze the data

In conclusion, we have developed the iMarmot database, which serves as a central portal for marmot species. iMarmot stores the biological information, genome sequences, gene information, synteny blocks, orthologous of three marmots, SNP data of five marmots and transcriptomic data from various hibernators and plagues hosts (Fig. 4). As iMarmot is a manually integrated database, all the raw data collected/submitted would be reprocessed by our own customized pipelines. For doing this, different results may generate while compared to the original published papers. The processed data is further related the retrieval and analysis module. The retrieval and analysis module, including various query, visualization and analysis tools, execute the main functions of the iMarmot database (Fig. 4). We believe that this database will not only broaden the understanding of genetic mechanism underlying marmots’ biological features, but also attract more and more attentions on the conservation of endangered marmot species.

## Data Availability

The datasets generated and analyzed in the current study are freely available on the Download page of iMarmot database with the web link: http://www.marmotdb.org/MARDB/download/toIndex.

## Abbreviations

ADW: Animal Diversity Web
DEGs: Differentially Expressed Genes
GEO: Gene Expression Omnibus
GO: Gene Ontology
IUCN Redlist: International Union for Conservation of Nature Redlist
KEGG: Kyoto Encyclopedia of Genes and Genomes
NCBI: National Center for Biotechnology Information
Nr: Nonredundant protein database
Nt: Nonredundant nucleotide sequence database
